# Intra-operative ultrasound-based augmented reality guidance for laparoscopic surgery

**DOI:** 10.1049/htl.2017.0063

**Published:** 2017-09-11

**Authors:** Rohit Singla, Philip Edgcumbe, Philip Pratt, Christopher Nguan, Robert Rohling

**Affiliations:** 1 Department of Electrical and Computer Engineering, University of British Columbia, Vancouver, Canada V6T1Z4; 2 MD/PhD Program, University of British Columbia, Vancouver, Canada V6T1Z4; 3 Department of Surgery and Cancer, Imperial College London, UK, SW72BX; 4 Department of Urological Sciences, University of British Columbia, Vancouver, Canada V6T1Z4; 5 Department of Mechanical Engineering, University of British Columbia, Vancouver, Canada V6T1Z4

**Keywords:** biomedical ultrasonics, surgery, kidney, augmented reality, medical robotics, tumours, cancer, calibration, healthy parenchymal tissue, registration error, robot-to-camera calibration, robot-assisted partial nephrectomies, tumour excision, single navigation aid, surgical instrument tracking, kidney, endophytic tumour, laparoscopic surgery, intra-operative ultrasound-based augmented reality guidance

## Abstract

In laparoscopic surgery, the surgeon must operate with a limited field of view and reduced depth perception. This makes spatial understanding of critical structures difficult, such as an endophytic tumour in a partial nephrectomy. Such tumours yield a high complication rate of 47%, and excising them increases the risk of cutting into the kidney's collecting system. To overcome these challenges, an augmented reality guidance system is proposed. Using intra-operative ultrasound, a single navigation aid, and surgical instrument tracking, four augmentations of guidance information are provided during tumour excision. Qualitative and quantitative system benefits are measured in simulated robot-assisted partial nephrectomies. Robot-to-camera calibration achieved a total registration error of 1.0 ± 0.4 mm while the total system error is 2.5 ± 0.5 mm. The system significantly reduced healthy tissue excised from an average (±standard deviation) of 30.6 ± 5.5 to 17.5 ± 2.4 cm^3^ (*p* < 0.05) and reduced the depth from the tumor underside to cut from an average (±standard deviation) of 10.2 ± 4.1 to 3.3 ± 2.3 mm (*p* < 0.05). Further evaluation is required in vivo, but the system has promising potential to reduce the amount of healthy parenchymal tissue excised.

## 1 Introduction

In laparoscopic surgery, a surgeon must operate with a laparoscope and long rigid surgical instruments. The laparoscope provides a video feed, which is displayed via a monitor. When the surgeon looks at the display, the surgeon has a reduced field of view and limited depth perception. Even with advances in robot-assisted procedures, visualisation challenges persist. The consequence is that it makes soft tissue abdominal surgery difficult. For example, this happens when the surgeon excises small renal cell carcinoma masses.

Compared to the radical nephrectomy approach, a partial nephrectomy has several advantages including reduced patient pain, improved post-operative total renal function, reduced risk of renal insufficiency and proteinuria, and equivalent cancer outcomes [1]. In fact, it has been associated with improved survival over radical nephrectomy [2]. Two goals of partial nephrectomy are to minimise the amount of healthy kidney tissue excised and to remove the cancerous tumour in its entirety. These tumours can range in location, size, and depth with some being endophytic (those with significant volume lying subsurface). These tumour descriptors are part of what determines the tumour's RENAL nephrometry score [3]. This score is used to quantify the complexity of renal masses in order to guide management decisions. A high RENAL score is reflective of a difficult surgery [3]. Endophytic tumours have been shown to have a complication rate that is nearly five times higher than exophytic tumours (those with significant volume lying above the surface) [4]. To spare healthy tissue, surgeons attempt to minimise the surgical margin size they excise while performing the operation. Margin size has been shown not to impact the risk of cancer recurrence [5]. Minimal margin size is recommended as 5 mm [5]. With this tissue minimisation goal, there is also a time constraint under which the surgeon must operate. During excision, the kidney is clamped off from its blood supply and exceeding 25 min on clamp increases the risk of permanent kidney injury [6]. The surgeon must operate under this time constraint in a reduced sensory environment, where there is limited or a lack of haptic feedback, and poor visualisation. Tasks requiring fine motor skills or complex manipulation are difficult. This forces the surgeon to make certain intra-operative surgical decisions in haste, such as determining the depth at which to cut underneath the tumour.

Using medical imaging, such as pre-operative CT or laparoscopic ultrasound (US), the surgeon can adjust their operational plan. The use of imaging assists with the understanding of tumour boundaries and depth, and in estimating the margins to cut. With regards to CT however, the kidney has been shown to move a significant amount between the pre-operative scan and the operation itself [7]. US is an appealing real-time and low-cost modality which provides the necessary imaging depth for subsurface structures. It is, however, user dependent. Further, neither CT nor US is present through the actual excision into the kidney; while they inform, they do not guide. If it were possible to have imaging-based data throughout the surgery, in the form of augmented reality (AR), it would assist the surgeon in overcoming the numerous operating challenges they face. In recent survey of urologists, 87% felt AR has potential to be used for navigation and is of interest to the medical community [8].

There has been significant work in the field of AR for laparoscopic surgery over the past decade. Ukimura and Gill reported one of the first uses of AR in urological procedures, presenting 3D visualisation for both laparoscopic partial nephrectomy and radical prostatectomy [9]. They reported that the use of AR is feasible and improved the surgeon's anatomical understanding [9]. Teber *et al.* presented an AR system that leveraged cone-beam CT intra-operatively and multiple radio-opaque needles to track the organ in real-time [10]. This work recently reached clinical use, as Simpfendorfer *et al.* reported the successful use of AR for intra-operative guidance in partial nephrectomies on humans with complex or endophytic tumours [11]. However, this guidance requires the use of cone-beam CT and introduces radiation, limiting its broad use. Wild *et al.* have similarly used multiple near-infrared fluorescent markers for intra-operative registration and AR [12]. These markers are promising as they are metabolisable but the use of multiple markers into the organ is still required. Su *et al.* showed it is feasible to register pre-operative CT data to the laparoscopic scene, achieving 1 mm registration accuracy [13]. However, this required a manual initial alignment and was not real-time.

On the use of US imaging for AR guidance, Cheung *et al.* presented a visualisation platform using fused video and US [14]. The platform used electromagnetic tracking of an US transducer for both 2D and 3D visualisation. This system was accurate to within 2.38 ± 0.11 mm, and showed no significant improvement in excision time [14]. While the planning time was reduced, this stage is often untimed as the kidney is unclamped. Pratt *et al.* have displayed 2D US images within the surgical scene using computer-vision-based tracking of the US transducer but did not explore its potential use during excision [15]. In a similar vein, Zhang *et al.* have extended the idea of transducer tracking with computer vision to non-planar transducer [16]. Neither work however explores guidance during excision. One work that investigated US imaging for AR guidance during excision lacked clinically acceptable accuracy [17].

A recent review by Bernhardt *et al.* further covers the extensive work done in the laparoscopic AR field and outlines the numerous challenges that remain – from pre-operative to intra-operative registration, tracking accuracies, and depth perception [18]. Bernhardt *et al.* identify that there are outstanding requirements such as AR validation, reliability, and usability [18]. There remains a need to leverage non-ionising, real-time intra-operative imaging and provide surgical guidance throughout the excision stage.

This work contributes to the field by addressing the challenges of intra-operative augmentation of deformable organs like the kidney. It presents new augmentations for use in laparoscopic partial nephrectomy based off intra-operative US imaging. This preliminary work presents a proof-of-concept AR guidance system that combines US, computer-vision-based tracking and kinematics-based tracking to provide continuous real-time guidance during tissue excision. The standalone prototype system is composed of a surgical navigation aid and an US transducer, requiring no extrinsic tracking hardware. It uses the da Vinci surgical system^®^ (Intuitive Surgical, Sunnyvale, CA, USA) as a development and testing platform. The navigation aid is fixed relative to the tumour, tracks the local surface, and allows guidance to occur despite local tissue deformation. Biomechanical simulations are performed to verify this fixed relationship between navigation and tumour. A model of the tumour is created from an US scan tracked relative to this navigation aid. Using this model and the tracked instruments, four different augmentations are rendered. These include a proximity alert, an orientation cue, a virtual viewpoint, and a virtual projected path of the instruments. The different components and the system as a whole are evaluated for their contributions of error. Further, the prototype system is evaluated for its utility in simulated robot-assisted partial nephrectomies with an expert surgeon. The excised specimens are examined for reductions in simulated healthy tissue and the depth of cut. Qualitative experience is reported.

## 2 Methods

The system uses a 3D-printed navigation aid with 10 mm long barbed legs and a unique attachment point designed for the Pro-Grasp™ instrument [19]. It is designed in Solidworks (Solidworks, Waltham, MD, USA) and can be printed entirely in plastic (Proto3000, Vaughn, ON, USA). Due to the small size of the navigation aid, it can be dropped into the patient via a 12 mm trocar. The unique attachment point allows the navigation aid to be picked up in a repeatable and accurate manner. The barbed legs allow the navigation aid to be inserted into the renal cortex of the kidney, where it remains approximately fixed relative to the tumour. The navigation aid has a black-and-white grid of circles 3D printed on one face. This allows the navigation aid to be tracked using the same method described by Pratt *et al.* which has been shown to work reliably in vivo [15]. Using a single camera channel of the stereo high-definition laparoscope, a circle detection algorithm, implemented in OpenCV, is run to find the centroids of each circle on the grid. Subsequently, using the known planar geometry of the grid, its pose is estimated in relation to a calibrated camera. To increase computational speed, a motion estimation method is used to predict the search region [15].

**Fig. 1:**
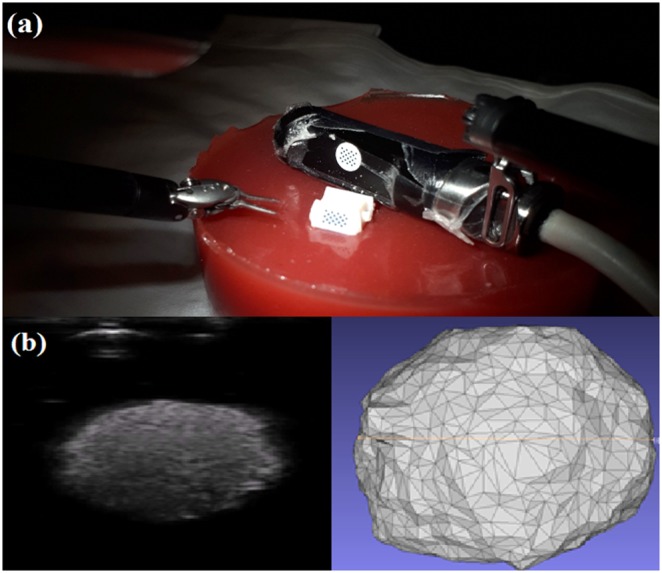
Overview of ultrasound-based tumour model generation *a* Simulated surgery setup, showing the navigation aid inserted into the phantom, and tracked US scan being performed *b* 2D US images (left) create a 3D tumour model (right)

The US transducer used is a custom ‘pick-up’ transducer created for robot-assisted surgeries [19]. The transducer has a 28 mm linear array and a center frequency of 10 MHz. It is compatible with analogic US machines (Analogic, Peabody, MA, USA). This transducer has the same repeatable attachment point design as the navigation aid, increasing surgeon autonomy when scanning the kidney. Schneider *et al.* showed that this transducer has a grasping repeatability within 0.1 mm in all axes, and within 1° for roll, pitch, and yaw [19]. The transducer has a KeyDot^®^ optical marker (Key Surgical, Eden Prairie, MN, USA) with a similar grid of circles that is also tracked in the same vision-based manner as the navigation aid. With this tracked transducer, an US volume is reconstructed relative to the navigation aid. The system setup during this stage is shown in Fig. 1. Volumes are manually segmented and surfaces are extracted using ITK-Snap as in Fig. 1. [20]. While this manual segmentation takes <5 min, this step occurs during the planning stage where the kidney is unclamped and there is no time constraint.

To assess the assumption of a fixed spatial relationship between the navigation aid and the tumour, biomechanical modelling is performed in ANSYS (ANSYS, Pittsburgh, PA, USA). This models the navigation aid's movement in a kidney during an US scan. The kidney is modelled as a 50 × 50 × 50 mm cube. The tumour is 20 mm in diameter and placed 20 mm within the kidney. The navigation aid is above the tumour, and the US transducer is placed 10 mm from the navigation aid's edge. Input parameters to the simulation include force applied by US transducer, leg length of the navigation aid, and kidney stiffness. Using a calibrated force sensor, the average maximum applied force for three complete US scans of phantoms is found to be 0.7 ± 0.3 N. Thus the forces of 0.1, 0.5, and 1.0 N are evaluated. The leg length is varied between 0, 5 and 10 mm. As Grenier *et al.* reports different cortical and medullary elasticities for in vivo human kidneys (15.4 ± 2.5 and 10.8 ± 2.7 kPa, respectively), simulations are performed using each average elasticity [21]. The distance between the theoretical tumour center (20 mm below the aid, regardless of pose) and the actual center is calculated.

The system is developed and evaluated on the da Vinci Surgical System. Through the research application programming interface of the da Vinci from Intuitive Surgical, the surgical instruments can be tracked relative to the laparoscope. For the simulated surgeries, kidney phantoms are created from polyvinyl chloride using Super Soft Plastic (M-F Manufacturing, Fort Worth, Texas, USA) and red dye. Spherical inclusions ranging in 10–30 mm of diameter are placed at a depth of ∼20 mm. Additionally, they are dyed black for ease of post-operative analysis. The phantom's elastic modulus is tabulated to be 15 kPa, matching what is reported for cortical tissue [21]. The gold standard for the tumour volume is determined by measured weight and known density from construction.

## 3 Calibration and total system error

**Fig. 2:**
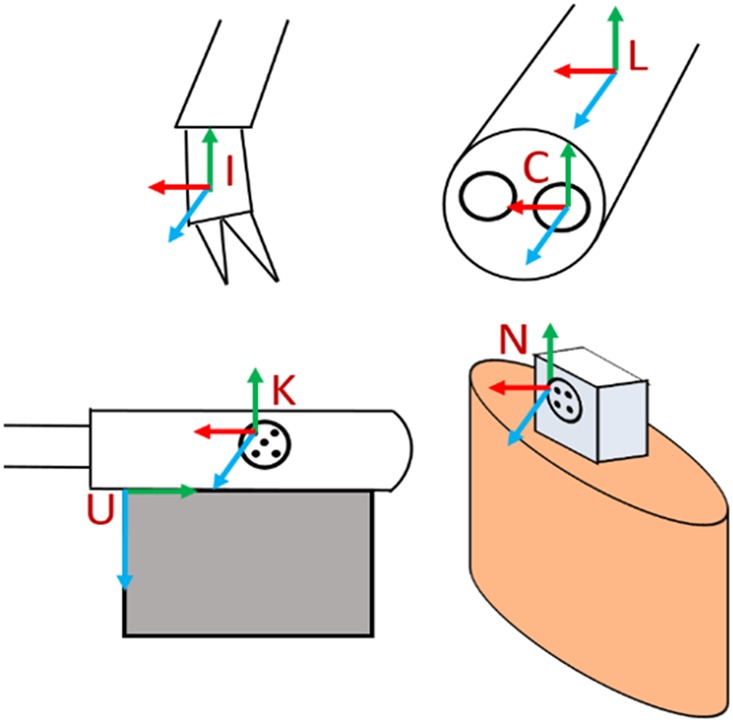
Illustration of the coordinate systems. The da Vinci tracks the instrument coordinate system, I, and the laparoscope coordinate system, L. The calibrated camera coordinate system, C, is different than L. The ultrasound coordinate system, U, is calibrated in relation to the KeyDot marker, K. The navigation aid coordinate system, N, is found on the barbed marker inserted into the soft organ

The stereo laparoscope is calibrated using Zhang's method implemented in OpenCV [22]. Only one camera is used for tracking, and no stereo triangulation is performed. Hereafter, camera refers to the left channel of the stereo laparoscope. The reprojection error of camera calibration is 0.4 pixels. The transformation from US image to the KeyDot^®^ on the US transducer was previously calibrated geometrically and found to have a pinhead reconstruction accuracy of 0.9 mm [17]. The same calibration is used here. With both a calibrated US transducer and a camera, it is possible to reconstruct a 3D US volume and create a tumour model. However, further calibration is required to bring the instruments and the model into the navigation aid's coordinate system. This is because the coordinate system of the laparoscope as tracked by the da Vinci differs from the one of the calibrated camera used for pose estimation of the marker, as illustrated in Fig. 2.

Denoting *^A^T_B_* to be a coordinate system transformation from coordinate system *B* to coordinate system *A*, the following transforms are present: the transformation from the surgical instrument to the laparoscope (*^L^T_I_*); the transformation from the laparoscope to the calibrated camera (*^C^T_L_*); and the transformation from the camera to the navigation aid (*^N^T_C_*). The first transformation is provided by the da Vinci tracking software, and the third is estimated using the computer-vision method described previously. Each of the coordinate systems referred to here is illustrated in Fig. 2. To solve for *^C^T_L_*, the tracking ability of the da Vinci instruments, known to be accurate to ∼1.0 mm, is used. By moving the navigation aid to different poses, and touching the known calibration grid with the surgical instrument, paired points in the camera coordinate system and in the laparoscope coordinate system are collected. Then, using a least-squares optimisation method, the unknown *^C^T_L_* is determined [23]. For this, 23 pairs of points are collected. Twelve pairs are used to calculate the transform, and the average fiducial registration error (FRE) is reported. The remaining 11 points are used to evaluate the target registration error (TRE).

**Fig. 3:**
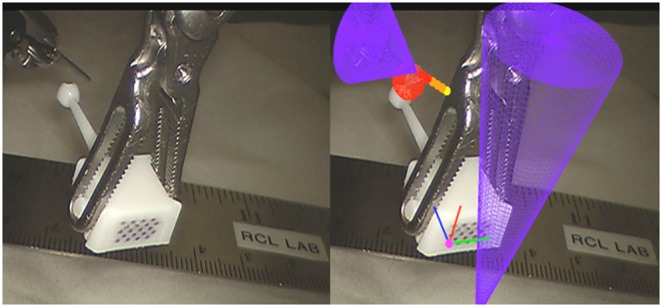
A comparison of the view without AR (left) and with AR (right). Red mesh model appears within 1 mm of ground truth ball tip, and AR overlays appear within 1 mm of ground truth. Purple cones are renderings of the tracked surgical tools

To evaluate the total system error, a modified navigation aid is used. This aid, seen in Fig. 3, includes a ball of 3 mm in diameter that extends outwards from the back. The ball itself has slots designed so that when the surgical instrument grasps the ball tip, the instrument tip and ball tip are coincident. System error can then be determined by comparing the known center from the optically tracked navigation aid and its associated known geometry and the instrument's location.

(1) $$P_N ={}^NT_C \times ^C\!\!T_K \times ^K\!\!T_U \times P_U\eqno\lpar 1\rpar $$

(2) $$P_N = {}^N\!T_C \times ^C\!\!T_L \times ^L\!T_I \times P_I\eqno\lpar 2\rpar $$

This error calculation can be represented by (1) and (2). Here, (1) takes a 2D pixel in the US (*P_U_*), transforms it from US to the KeyDot^®^ (*^K^T_U_*), to the camera (*^C^T_K_*), and then into the aid's coordinate system (*^N^T_C_*). This is used to generate the 3D US volume prior to segmentation. By comparing the segmented model's centroid to the known ground truth, it is possible to evaluate the error in vision-based tracking, reconstruction and segmentation. Subsequently, (2) is the 3D location of the instrument (*P_I_*) transformed to the laparoscope (*^L^T_I_*), to the camera (*^C^T_L_*) and into the navigation aid's coordinate system. By comparing the instrument's location to the ground truth, it is possible to evaluate the tracking error.

To evaluate these errors, the modified navigation aid is held by an instrument in a water bath at room temperature. The ball tip is scanned, reconstructed, and segmented. The navigation aid is moved to ten poses, still held by the instrument. This makes *P_U_ = P_I_*. The Euclidean distance between instrument's location and the ground truth centre is calculated in each pose, and the average is reported. Fig. 3 shows a side-by-side comparison with and without the resulting AR guidance using the modified navigation aid.

## 4 AR overlays and user study

As described, the surgeon must operate under a time constraint while minimising amount of healthy tissue excised. Owing to this, it is impractical to develop nuanced augmentations that cannot be quickly interpreted. While high fidelity overlays may be convincing, they are limited in utility if they are not intuitive and informative. With this design consideration, four different simple augmentations are proposed.

**Fig. 4:**
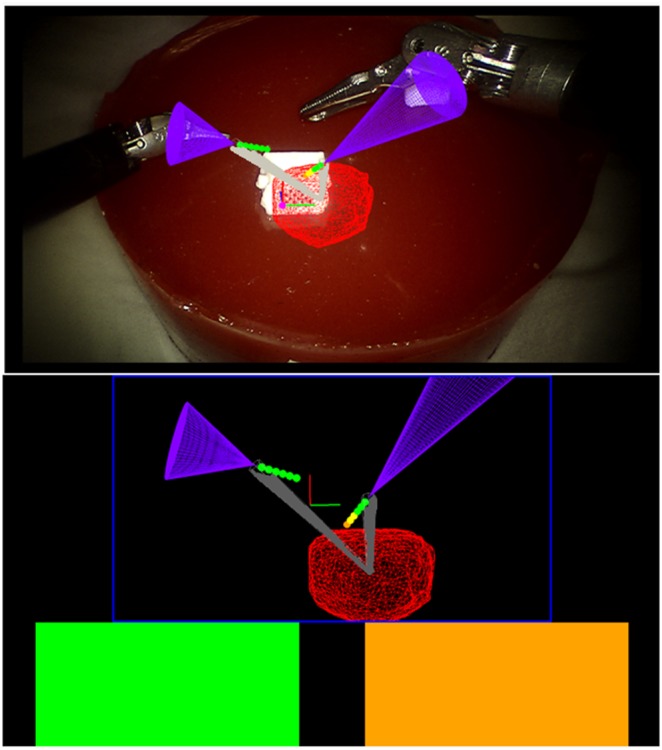
Augmentations presented to the surgeon in TilePro^®^. Tumour is seen in red, and virtual tools are in purple. Top shows the projected path with spheres. Bottom shows the traffic lights and virtual viewpoint. Compass is seen in grey in both views

The first is the *traffic light*: a colour-coded proximity alert of the instrument's distance to the tumour's surface shown as coloured blocks to the surgeon (Fig. 4). The surgeon sets four ranges of distance of the instrument to the tumour's surface. From these ranges, the alert flashed red, yellow, orange, or green. For this work, the ranges are <2.5 mm, between 2.5 and 3.5 mm, between 3.5 and 5.0 mm, and beyond 5.0 mm. A traffic light is provided for each of the two surgical instruments.

The second is the *compass*: a conical overlay that provides as an orientation cue for the surgeon. As the tumours in this work are endophytic, it is important to know the relative orientation of the tumour to an instrument at any given time, particularly if the instrument is behind the tumour. A grey cone pointing from the instrument to the tumour's centre is provided, with the cone's height equal to instrument to tumour distance (Fig. 4).

The third is the *instrument projected path*: a virtual extension with spheres of known diameter and spacing, set by the surgeon. In Figs. 3 and 4, the spheres are all set at 1 mm apart, with 1 mm diameters. The functionality of the traffic lights are combined with the spheres, allowing the surgeon to gauge the distance of their instrument to the tumour should they continue in their current pose. The fourth overlay is a *virtual viewpoint:* the projected virtual scene from a virtual camera placed 50 mm away from the tracked aid, facing perpendicular to the KeyDot^®^ (Fig. 4). Treating the aid as a planar approximation of the local surface, the surgeon can then see tumour depth from the surface virtually (Fig. 3). The augmentations are all displayed using the TilePro^®^ functionality of the da Vinci surgical system, rather than interrupting the surgeon's normal video feed. This circumvents the inherent lag in capturing a video feed, processing it, and feeding it back into a monitor. It also refrains from occluding the surgeon's primary video feed. All four augmentations are presented to the surgeon at the same time. The surgeon has the choice to refer to any of the augmentations at any given time. To provide real-time guidance, the augmentations leverage a signed distance field. This field is computed after the tumour model is generated from US, and incorporates a 10 mm margin from the tumour surface in each axis. It reduces the complexity of calculating the distance of a given instrument to the closest point on the tumour surface to being a look up table. It furthers captures irregularities in model topography, allowing for precise augmentations. This is particularly beneficial when the model is complex or contains additional structures.

To evaluate the clinical utility of the guidance, simulated partial nephrectomies are performed by an expert urologist. Each nephrectomy included an entirely endophytic tumour and is rated as having a RENAL score of 10×. An expert urologist with over 10 years of experience, and trained to operate with the da Vinci, completed 18 partial nephrectomies, nine using the system, and nine with laparoscopic US only. The surgeon is given the modified aid for training with AR. They are given a practice surgery using only US. For all surgeries, the excision times, excised specimen volume, margin status, and the depth at which they cut under the tumour, relative to the tumour itself, are recorded. Margin status indicates whether a positive or negative margin occurred, where a positive margin is defined as slight tumour exposure in the specimen (microscopic) or visible portions of tumour remaining in the kidney (gross). Volumes are determined by specimen weight and known density. To account for varying tumour depth, the top layer of parenchyma above the tumour is removed and the specimen re-weighed. Both the total volume and this adjusted volume are reported. Depth of cut is determined by US imaging of the excised specimens. For qualitative feedback, the surgeon completed a Likert-scale questionnaire adapted from the System Usability Scale after each surgery [24]. After all the surgeries are completed, the surgeon is given open-ended questions to answer about their experience using the AR system. A two-tailed paired *t*-test is performed for statistical significance with a power of 0.05. The Holm–Bonferroni correction is used to account for multiple comparisons.

## 5 Results

**Fig. 5:**
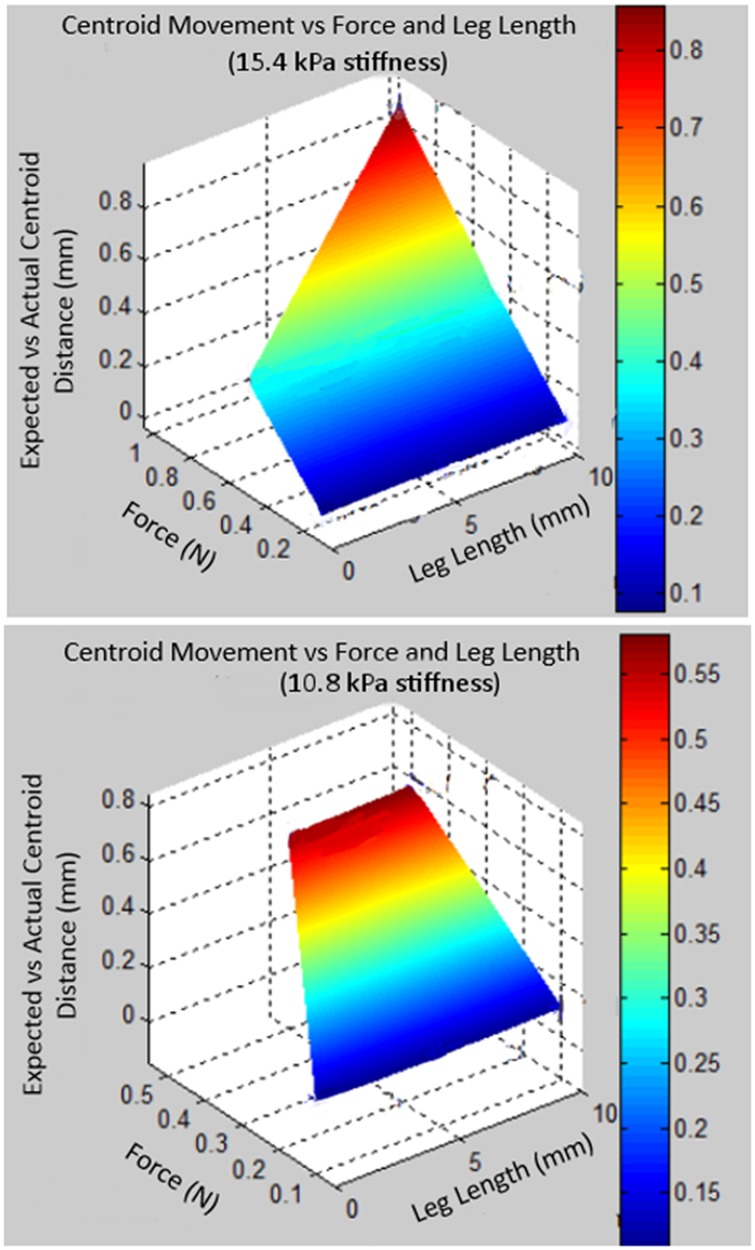
Simulation results of navigation aid's relationship compared with the tumour centroid. Euclidean distance between the expected and actual centroid position is plotted against force (N) and leg length (mm). Top graph is for a kidney cortical stiffness of 15.4 kPa. Bottom graph is for a kidney medullary stiffness of 10.8 kPa

For all finite-element simulations, which modelled the navigation aid relative to a tumour under different forces and stiffness, the distance between theoretical location of the tumour's centre and simulated tumour centre never exceeded 1 mm. From this, the rigidity assumption for the navigation aid results in an error in estimating kidney tumour location of no greater than 1 mm. Fig. 5 plots this error against the force and leg length and for both elasticity values.

The average ground truth tumour volumes excised with AR were 1.9 ± 0.4 cm^3^, compared to the average segmented tumour volumes of 2.7 ± 0.7 cm^3^. The average radius of the segmented volumes was 0.9 ± 0.3 mm larger than the ground truth. This indicates that the segmented models on average slightly over-estimated the tumours.

The FRE in calibrating the laparoscope coordinate system to the camera coordinate system (*^C^T_L_*) was 0.8 ± 0.3 mm using 12 pairs of points. The TRE for 11 different pairs was 1.0 ± 0.4 mm. The working volume covered was 45 × 30 × 50 mm. The total system error over ten poses was 2.5 ± 0.5 mm when comparing the instrument location, while holding the ball tip, against the ground truth centre. When compared against the segmented centre, the error was 1.4 ± 0.5 mm.

**Fig. 6:**
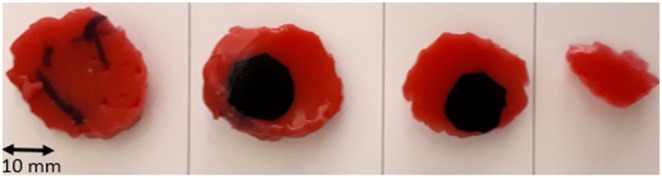
Cross section of a tumour excised with AR guidance. Slice closest to kidney surface on the left, farthest on the right. Each slice is ∼5 mm in thickness

The quantitative results of the surgeries performed are summarised in Table 1. These initial results show that, with no statistically significant difference in excision time, the surgeon was able to excise significantly less healthy tissue with AR guidance than without. Notably, the tumours excised with US and AR were not significantly different in volume, nor was there a significant difference in positive margin rate. However, the two positive margins with US were gross margins that left significant amounts of tumour behind. The single positive margin achieved with AR was microscopic, with no visible tumour left behind. Importantly, with AR, the surgeon was able to reduce the depth at which they cut past the tumour significantly, going from ∼10 to 3 mm. Fig. 6 shows an example specimen's cross sections excised with AR.

**Table 1:** Quantitative results of simulated partial nephrectomies as average and standard deviation. Bold indicates statistical significance (*p* < 0.05) of AR compared to the US only

Metric (mean ± SD)	US (*n* = 9)	AR (*n* = 9)
excision time, s	203 ± 30	257 ± 50
tumour volume, cm^3^	2.4 ± 1.0	1.9 ± 0.4
total excised specimen volume, cm^3^	30.6 ± 5.5	**17.5 ± 2.4**
adjusted excised specimen volume, cm^3^	22.1 ± 5.2	**10.6 ± 2.1**
depth cut under tumour, mm	10.2 ± 4.1	**3.3 ± 2.3**
positive margins (/9)	two gross	one microscopic

Qualitatively, the surgeon found the system easy to use, was confident in the system, and understood where he was spatially relative to the tumour. The surgeon reported that he imagined most people would learn how to use the system quickly, and that it was not cumbersome nor unnecessarily complex. Importantly, the surgeon felt the system met his guidance needs during surgery. When asked to rank the AR overlays in order of most to least preferred, the expert indicated that he strongly preferred the projected path, then the traffic lights, the compass, and finally the virtual viewpoint.

## 6 Discussion

This work presents a novel prototype AR guidance system for laparoscopic surgery using intra-operative US imaging. The navigation aid was simulated using finite-element analysis and, relative to the tumour, did not deviate beyond 1.0 mm in comparison to the expected distance. This is adequate for the purposes of providing guidance in the soft kidney. The total system error of 2.5 ± 0.5 mm is acceptable when considering a 5 mm margin as the standard of care. This prototype system meets the accuracy requirement to be useful in guidance. Since the printing precision is 14 μm, the known geometry of the aid can also provide intra-operative AR validation. It can be picked up by the surgeon reliably at any time and used to verify the accuracy of the guidance provided. It can inform whether re-calibration is required intra-operatively.

Overall, the AR was beneficial in resecting the lateral edges of the specimen. It was informative in determining the plane to cut underneath the tumour and was considered essential in guiding the deep resection through tissue. The AR was noted as being predictable when it would and would not appear (due to occlusion of the navigation aid). This was frustrating but beneficial as the surgeon could understand why no guidance was presented at times and how to resolve it. This line-of-sight issue could be mitigated with the use of multiple navigation aids, added contemporaneously during excision. Using the projected path and its incorporated traffic light, the surgeon adopted a ‘check and go’ strategy, a minor modification to his traditional excision approach where he paused during cutting and checked his tool's surroundings. At various points where the spheres were hard to see or his instruments were occluded, the proximity alerts were still used as a proxy. Counter-intuitively, this modified strategy did not significantly increase the excision time. However, the mean did increase which may be due to a learning curve effect that may decrease over time. With respect to the virtual viewpoint, the surgeon elaborated that, although useful in concept, it is difficult to interpret quickly and mentally register the scene while under a time constraint. In an untimed stage of the surgery, for example the planning stage where the renal hilum is not clamped and kidney perfusion is nominal, a virtual viewpoint may be beneficial. The projected path and traffic light overlays provide limited depth perception. The projected path, which copies the laparoscopic view and renders on top of it, is created using a single camera feed, contrary to the surgeon's 3D stereoscopic video feed. This can be improved using TilePro^®^ to provide a 3D stereo AR. The four augmentations were however compared all at once, rather than separately or in pairs. This was done as each augmentation serves a different function. However, investigation into the human–computer interfaces and identifying the optimal augmentation is warranted. One limitation of the evaluation is that the tumours were of different radii, potentially affecting the excised volumes. Having varying radii does reflect the variance in tumours in practice, and the tumour volumes were not found to be significantly different, However, the tumour volumes were not statistically significantly different between groups, as reflected in Table 1,

While the study is small with a single user performing 18 surgeries, it does demonstrate the feasibility of using tracked US to create continuous guidance with encouraging results. The surgeon was able to use the AR system to reduce significantly the amount of healthy tissue excised, at no increase to excision time, and with a reduced risk of cutting into the collecting system. Such guidance may enhance how the surgeon operates, when excising around the tumour, and beneath it.

## 7 Conclusion

This work shows that the use of intra-operative US for guidance can significantly improve the depth at which a surgeon cuts underneath a tumour, leading to a reduction of healthy tissue excised. The work presents four augmentations of varying fidelity. Of these, the surgeon used the projected path which mimicked his real environment the most. Such guidance can mitigate the uncertainty in which the surgeon operates. Future work includes in vivo testing to evaluate robustness, and further experimentation with different augmentations to optimise the user experience. That said, with minimal additional hardware (a single low-cost navigation aid), feasible intra-operative US-based guidance during excision is possible and can be made widely available for laparoscopic surgery.
